# Antibiotic Resistance to Escherichia coli among Urine Culture-Positive Patients in a Tertiary Care Hospital in Nepal: A Descriptive Cross-sectional Study

**DOI:** 10.31729/jnma.5545

**Published:** 2021-01-31

**Authors:** Anu Kushwaha, Khilasa Pokharel, Anuj Raj Kadel

**Affiliations:** 1 Department of General Practice and Emergency, Kathmandu Medical College and Teaching Hospital, Sinamangal, Kathmandu, Nepal; 2 Department of Microbiology, Kathmandu Medical College and Teaching Hospital, Sinamangal, Kathmandu, Nepal; 3 Department of Medical Education, Kathmandu Medical College and Teaching Hospital, Sinamangal, Kathmandu, Nepal

**Keywords:** *antibiotic*, *drug resistance*, *urinary tract infections*

## Abstract

**Introduction:**

Urinary tract infection is treated by the antibiotic sensitivity pattern of uropathogens in our population. Present infection cases have been showing an increase in resistance to the current first-line of antibiotics. The objective of this study is to determine the resistance of antibiotics in Escherichia coli in a tertiary care center.

**Methods:**

This is a descriptive cross-sectional study done in Kathmandu Medical College Teaching Hospital from October 2018 to February 2019. The sample size was calculated, and convenient sampling was done. Patients with urine culture positive (i.e., Colony-forming unit >105) were included in the study. All positive samples were tested for antibiotics sensitivity and resistance. The resistance to the antibiotics was recorded using Statistical Package for Social Sciences version 20. Point estimate at 95% confidence interval was calculated along with frequency and proportion for binary data.

**Results:**

Out of 100 samples, the highest number of organisms isolated was Escherichia coli, 71 (71%). Escherichia coli showed the highest resistance to drugs like Norfloxacin, 37 (52.11%), Amoxicillin-Clavulanic acid 37 (52.11%), followed by Co-trimoxazole 32 (45.1%), Ceftriaxone, 24 (33.8%), and Ciprofloxacin 23 (32.3%).

**Conclusions:**

Escherichia coli showed the highest resistance to commonly used antibiotics like Norfloxacin, Amoxicillin-Clavulanic acid, Co-trimoxazole, Ceftriaxone, and Ciprofloxacin.

## INTRODUCTION

Urinary tract infection (UTI) is defined as the presence of pathological bacteria in the urinary system from the kidney to the urethra. After respiratory tract infection, urinary tract infection is the most common infection in our society.

UTI in the genito-urinary tract without any instrumentation is called uncomplicated UTI. In the structurally and functionally abnormal urinary tract and associated with instrumentation, it is called complicated UTI.^1^ Severe form of UTI is due to Escherichia coli (E. coli).^2^ Klebsiella, Proteus, Pseudomonas, Staphylococcus, Enterobacter, and Enterococci are other organisms causing UTI. There is an increased risk of developing antimicrobial resistance due to the unregulated use of antibiotics worldwide.^3^ Empirical treatment for UTI in patients has to be formulated time and again, given the increasing tendency of antibiotics resistance in microorganisms.

This study aims to find the prevalence of urinary tract infections caused by E. Coli among urine culture-positive patients and identify which antibiotics E. Coli has developed resistance against.

## METHODS

A descriptive cross-sectional study was carried out in the Clinical Microbiology Laboratory of Kathmandu Medical College and Teaching Hospital. Ethical approval was taken from the Institutional Review Committee (IRC), Ref no:2311201812, KMCTH. All urine culture-positive patients were included in the study, and patients with culture-negative are excluded from the study.

An antibiotic sensitivity test of E. coli isolated from clinical specimens against different antibiotics was performed on Muller Hinton Agar (MHA) by the Kirby-Bauer method's standard disk diffusion technique.^4^

Convenient sampling was done, and the sample size was calculated using the formula:

$$\begin{array}{ll} \text{n} & ={\text{Z}}^{\text{2}}\times \text{p}\times \text{(}1-\text{p)}/{\text{e}}^{\text{2}}\\ & ={\left(1.96\right)}^{2}\times 0.5\times (1-0.5)/{\left(0.1\right)}^{\text{2}}\\ & =96 \end{array}$$

Where,

n = Sample size
p = 50%
q = 1-p
e = Margin of error, 10%
Z = 1.96 at 95% Confidence Interval (CI).
100 samples were taken.

Data analysis was done using Statistical Package for the Social Sciences-20 version.

## RESULTS

Out of the 100 urine culture samples which tested positive for an organism, Escherichia coli was isolated in 71 (71%) of them. E. coli showed resistance to drugs like Norfloxacin, 37 (52.11%), Amoxicillin-Clavulanic acid, followed by Co-trimoxazole, 32 (45.1%), Ceftriaxone, 24 (33.8%) and Ciprofloxacin, 23 (32.3%) (Table 1).

**Table 1:** Antibiotic resistance to E. coli.

Antibiotics name	Resistance seen in urine culture samples in which E. Coli was isolated n (%)
Amikacin	9 (12.67)
Ampicillin+Sulbactam	2 (2.82)
Amoxicillin+Clavulanic acid	37 (52.11)
Amoxicillin	0 (0)
Azithromycin	7 (9.86)
Clavulanic acid	2 (2.82)
Ceftazidime	1 (1.41)
Cefsulodine	29 (40.8)
Cefixime	1 (1.41)
Ciprofloxacin	23 (32.3)
Chloramphenicol	1 (1.41)
Co-trimoxazole	32 (45.1)
Cloxacilln	0 (0)
Ceftriaxone	24 (33.8)
Cefotaxime	2 (2.82)
Gentamicin	2 (2.82)
Imipenem	4 (5.63)
Meropenem	6 (8.45)
Nitrofurantoin	0 (0)
Norfloxacin	37 (52.11)
Polymyxin B	0 (0)
Piperacillin+Tazobactam	1 (1.41)
Tobramycin	3 (4.23)
Total Samples	71 (100)

Hence highest resistance was present for Norfloxacin and Amoxicillin-Clavulanic acid. No resistance was seen for Amoxicillin, Cloxacillin, Nitrofurantoin, and Polymyxin B.

## DISCUSSION

This study evaluated the antibiotics resistance pattern of E. coli. In this study, the maximum number of patients in the age group of 21-30 was UTI. This may be attributed to the fact that 21 to 30 is a sexually active age group. Similar patterns were also observed in studies done by Mohamad Akram et al. in AligarhIndia.^3,4,5^

The higher incidence of UTI among females is likely due to anatomical and hormonal variation among the two sexes, making the female gender more susceptible to the infection. This discrepancy due to gender was consistent with studies done in another center.^5,6^ Unsurprisingly, E. coli was the commonest organism isolated from the urinary samples.^7–9^

E.coli showed high resistance to Amoxyclav, Norfloxacin, Cefsulodin, Ceftriaxone, Ciprofloxacin, and Co-trimoxazole low resistance to Gentamicin, Meropenem, Imipenem, and Amikacin. These results were almost similar to the findings reported by Oge Kale Olunalan Timothy et al. and SiddiqueM et al.^7,8^

Uropathogens are increasingly becoming resistant to several commonly used antibiotics. Thus, resistance and sensitivity patterns of microorganisms causing infectious diseases, including UTI, are necessary to provide a proper guideline. This increased resistance may be a consequence of unregulated antibiotics use and warrants strict rules and regulations on antibiotics’ consumption and prescription.

## CONCLUSIONS

E. coli was the most common bacteria isolated from urinary tract infection, followed by K. pneumonia. These uropathogens are increasingly becoming resistant to several commonly used antibiotics, controlled by formulating a proper guideline for treating physicians. This increased resistance may be a consequence of unregulated antibiotics use and warrants strict rules and regulations on antibiotics’ consumption and prescription.
